# Practice network-based care management for patients with type 2 diabetes and multiple comorbidities (GEDIMAplus): study protocol for a randomized controlled trial

**DOI:** 10.1186/1745-6215-15-243

**Published:** 2014-06-21

**Authors:** Kayvan Bozorgmehr, Joachim Szecsenyi, Dominik Ose, Werner Besier, Manfred Mayer, Johannes Krisam, Christian O Jacke, Hans-Joachim Salize, Ralf Brandner, Sandra Schmitt, Marion Kiel, Martina Kamradt, Tobias Freund

**Affiliations:** 1 Department of General Practice and Health Services Research, University Hospital Heidelberg, Voßstrasse 2, Gebäude 37, Heidelberg 69115, Germany; 2 Genossenschaft Gesundheitsprojekt Mannheim e G, Liebfrauenstrasse 21, Mannheim 68259, Germany; 3 Department for Medical Biometry, Institute for Medical Biometry and Informatics, University Hospital Heidelberg, Im Neuenheimer Feld 305, Heidelberg 69120, Germany; 4 Central Institute of Mental Health (ZI), Medical Faculty Mannheim, Heidelberg University, D6-5, Mannheim 69159, Germany; 5 InterComponentWare AG, Altrottstraße 31, Walldorf 69190, Germany

**Keywords:** Care management, Chronic care, Diabetes, Multi-morbidity, Primary care, Self-care

## Abstract

**Background:**

Care management interventions in the German health-care system have been evaluated with promising results, but further research is necessary to explore their full potential in the context of multi-morbidity. Our aim in this trial is to assess the efficacy of a primary care practice network–based care management intervention in improving self-care behaviour among patients with type 2 diabetes mellitus and multiple co-occurring chronic conditions.

**Methods/Design:**

The study is designed as a prospective, 18-month, multicentre, investigator-blinded, two-arm, open-label, individual-level, randomized parallel-group superiority trial. We will enrol 582 patients with type 2 diabetes mellitus and at least two severe chronic conditions and one informal caregiver per patient. Data will be collected at baseline (T0), at the primary endpoint after 9 months (T1) and at follow-up after 18 months (T2). The primary outcome will be the differences between the intervention and control groups in changes of diabetes-related self-care behaviours from baseline to T1 using a German version of the revised Summary of Diabetes Self-Care Activities (SDSCA-G). The secondary outcomes will be the differences between the intervention and control groups in: changes in scores on the SDSCA-G subscales, glycosylated haemoglobin A level, health-related quality of life, self-efficacy, differences in (severe) symptomatic hypoglycaemia, cost-effectiveness and financial family burden. The intervention will be delivered by trained health-care assistants as an add-on to usual care and will consist of three main elements: (1) three home visits, including structured assessment of medical and social needs; (2) 24 structured telephone monitoring contacts; and (3) self-monitoring of blood glucose levels after T1 in 3-month intervals. The control group will receive usual care. The confirmatory primary analysis will be performed following the intention-to-treat (ITT) principle. The efficacy of the intervention will be quantified using two-level linear regression stratified by type of medical treatment adjusted for baseline values on the SDSCA-G. Secondary analyses will be performed according to the ITT principle. In health economic evaluations, we will estimate the incremental cost-effectiveness ratios.

**Discussion:**

We hope that the results of this study will provide insights into the efficacy of practice network–based care management among patients with complex health-care needs.

**Trial registration:**

Current Controlled Trials ISRCTN 83908315 (ISRCTN assigned 25 February 2014).

## Background

The increasing prevalence of multiple, co-occurring chronic conditions constitutes a burden for contemporary health systems internationally [1] as well as in Germany [2]. Multi-morbidity is associated with worse health outcomes, more complex clinical management and increased health-care costs [3]. Several suggestions have been made regarding taking up the challenge of addressing this problem by reorganizing the delivery of chronic illness care [4,5]. On the basis of these concepts, care management interventions focusing on patients with multiple chronic conditions have been developed and evaluated [6]. *Care management* has been defined as ‘a set of activities designed to assist patients and their support systems in managing medical conditions and related psychosocial problems more effectively, with the aim of improving patients’ health status and reducing the need for medical services’ [7]. These interventions share four core elements [8]: (1) comprehensive assessment of patients’ medical and nonmedical needs and resources, (2) implementation and monitoring of individualized, evidence-based care plans, (3) coordination of services between providers of medical and social care and (4) enhancement of the self-management capabilities of patients and caregivers.

In the context of type 2 diabetes mellitus, the concept of ‘self-care’ (a synonym for self-management) is playing an increasingly important role in preventing complications and coping with the consequences of the condition [7]. Relevant interrelated self-care dimensions for patients with type 2 diabetes include adaptations of diet, exercise, self-monitoring of blood glucose (SMBG), foot care, medication adherence and smoking [9]. Diabetes typically occurs with or has other chronic diseases as consequences. Discordant comorbidities, such as depression [10], constitute an additional challenge for patients with diabetes to effectively conduct self-care activities.

Evidence suggests that, at least in the short run, self-care behaviour can be modified through self-management interventions [11]. However, it is important that interventions aimed at supporting patients to cope with diabetes and potentially co-occurring conditions (1) are person-centred (not disease-centred), comprehensive and embedded in the process of primary care within the health-care system and (2) take into account and mobilize community resources.

Although structured disease management programs (DMPs) are embedded in routine primary care for patients with type 2 diabetes in Germany [12], their ‘disease-centred’ design does not sufficiently take into account the challenge of comorbidity. For care management programs, positive effects on quality of care and patients’ quality of life have been reported. Still, though, the effect of care management interventions on health-care utilization and costs remains heterogeneous [7]. This finding is reinforced by a recent systematic review of complex interventions designed to improve outcomes in patients with multi-morbidity (including comorbidity) in primary care and community settings [13]. The review concludes that there is limited evidence on the care of patients with multi-morbidity, despite the prevalence of multi-morbidity and its impact on patients and health-care systems [13].

Few researchers have assessed the effects on patient-reported outcomes (PROs), such as self-care behaviour, self-efficacy, attitudes, health service utilization, quality of life and/or psychological indicators [14,15]. There are also few trials in which the investigators reported ‘patient-important’ outcomes related to death, morbidity (including hypoglycaemic events) and quality of life [16].

Care management interventions in the German health-care system have been evaluated, with researchers reporting promising results for patients with osteoarthritis [17], depression [18], chronic heart failure (CHF) [19] and multi-morbidities [6]. However, further studies are necessary to explore the full potential of care management interventions to improve outpatient diabetes care in Germany in the context of multi-morbidity. Care management is particularly challenging for small primary care practices (PCPs) to deliver. Primary care practice network (PCPnetwork)–based approaches might be a solution in this context, because they allow provision of intensified care from several practices, thus reducing the workload of smaller PCPs. These approaches in turn entail challenges regarding the flow of information between care managers, PCPs and other sectors in the delivery of relevant health-care services according to individual patients’ needs.

We have developed a complex PCPnetwork-based, health care assistant (HCA)–led, information technology (IT)–supported care management intervention based on an existing pilot project. It involves integrated telephone monitoring to improve diabetes care for patients with type 2 diabetes mellitus and multiple comorbidities. The study protocol of our trial includes documentation of the evaluation methods to be deployed during the ‘evaluation stage’ [20] of the complex intervention.

## Methods/Design

### Primary objective

**Figure 1:**
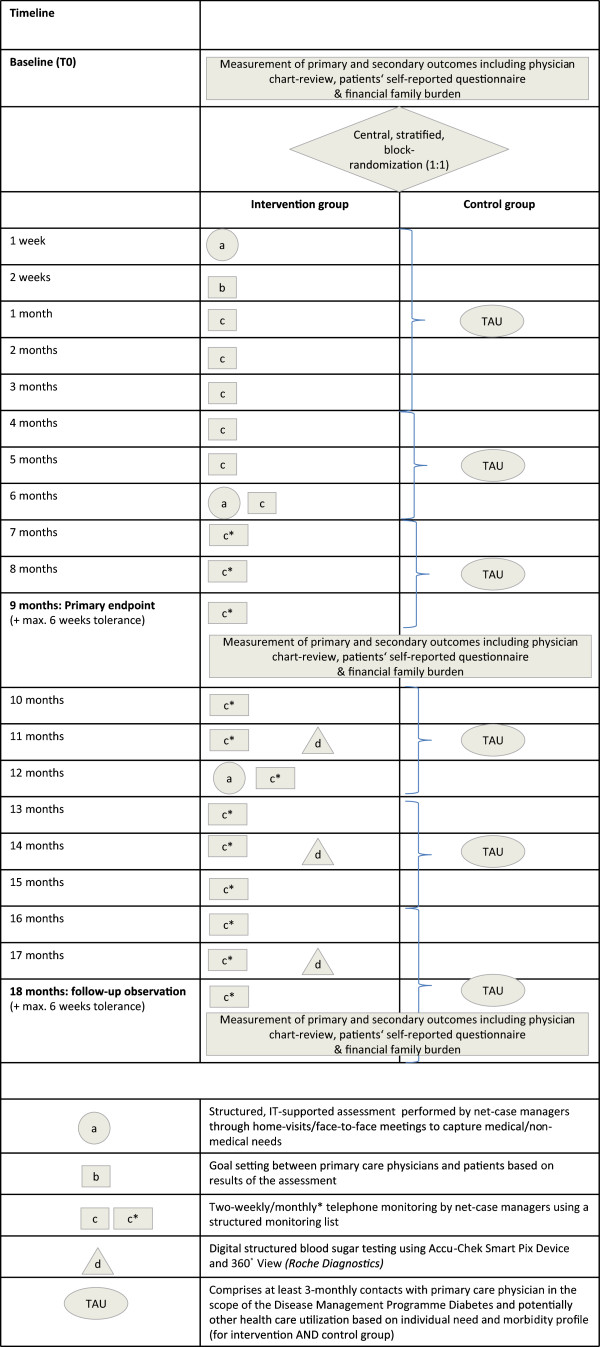
**Graphical illustration of the elements and the timeline of the GEDIMAplus trial.** Adapted from Perera *et al*. [21].

Our primary objective in this study is to assess the efficacy of a PCPnetwork-based, HCA-led, IT-supported care management intervention with integrated telephone monitoring for the improvement of self-care behaviour among patients with type 2 diabetes mellitus and two or more comorbidities. We aim to determine if there is a difference in changes in diabetes-related self-care behaviour after 9 months (T1) of intensified care management (as an add-on to usual care) compared to baseline (T0) between the intervention group, which will receive the Pathways to Change (PTC) intervention, and the control group (treatment as usual (TAU)), which will receive usual care only (Figure 1).

### Secondary objectives

We further aim to compare the groups (PTC vs. TAU) in the following aspects:

1. Change in diabetes-related self-care behaviour compared to T0 after an 18-month period (T2) with reduced intensity of care management and enhanced by SMBG, with *reduced intensity* referring to longer time lags between telephone monitoring contacts (Figure 1).

2. Change in diabetes-related self-care behaviour after 9 months (T1)/18 months (T2) compared to T0, adjusted for behaviour at baseline and other important factors reported in the literature (sex, age, marital status/domestic partnership, social status, migration status, type of comorbidity, perceived quality of health care, alcohol abuse and diabetes-related emotional distress).

3. Changes in each dimension of diabetes-related self-care behaviour (diet, exercise, SMBG, foot care and smoking) at T1/T2 compared to T0.

4. Differences in change of glycosylated haemoglobin A1c (HbA1c, %) at T1/T2 compared to T0 in the subgroup of patients with a HbA1c T0 value >7.5% (adjusted for the T0 HbA1c value).

5. Differences in the cumulative incidence of (severe) symptomatic hypoglycaemia at T1/T2 compared to T0 among those patients at risk for this event during the respective observation period.

6. Differences in change of health-related quality of life (HRQL) and self-efficacy at T1/T2 compared to T0

7. The distribution of potential health gains (that is, improvements in self-care behaviour, HbA1c percentage and HRQL) at T1 across socioeconomic groups to assess whether the intervention is equitable.

8. Differences at T1/T2 related to service utilization, direct and indirect costs of health-care provision during observation time (The financial family burden of informal care provided by patients’ relatives is particularly regarded within indirect costs).

9. The cost-effectiveness of the primary endpoint (change in self-care behaviour at T1).

### Study design

The study is designed as a prospective, 18-month, multicentre, two-arm, open-label, individual-level, randomized parallel-group superiority trial (RCT). Eligible participants will be randomly selected from among the aggregate patient population of 30 study centres (PCPs), and they will be asked for their consent to take part in the study. Participating patients will be given a pseudonym by the recruiting physician and only the pseudonym will be reported to the study central office (the Department of General Practice and Health Services Research, University Hospital Heidelberg (AMV)), where the patients will be centrally randomized on an individual level by a clinical monitor, who will act as the sole randomization authority. This person, who will not be involved in the care of the trial patients or analysis of data, will be responsible for allocation to the control arm (TAU) and the intervention arm (PTC) according to a predefined randomization schedule. As illustrated in Figure 1, the intervention will be provided in addition to usual care. Blinding of either patients or practice teams is not possible, owing to the character of the intervention. The T0 assessment of primary and secondary outcomes will be conducted prior to allocation of patients and after allocation at 9 months (primary endpoint: T1) and at 18 months (follow-up: T2) (Figure 1). Both the intervention and control groups will receive usual care. Usual care will consist of diabetes care within the scope of the German disease management program for type 2 diabetes mellitus, DMP Diabetes. Care provided for high-prevalence chronic comorbidities such as chronic obstructive lung disease COLD, heart failure and coronary heart disease (CHD) will also be organized within the scope of DMPs on a voluntary basis in the German statutory health insurance system [13]. Care provided for other diseases (chronic or acute) will be managed by PCP physicians individually based on personal experience, guidelines and/or patients’ preferences.

### Inclusion and exclusion criteria

#### Primary care practices

To function as a study centre, physicians based at PCPs must fulfil the following criteria: They must be specialized in general practice or internal medicine or as ‘medical practitioners’ (praktischer Arzt) and must function as ‘primary care physicians’ (Hausarzt) according to German regulations. Both group practices (Gemeinschaftspraxen) and individual practices are eligible to participate. Inclusion criteria for PCPs will be captured with a brief, individualized questionnaire. PCPs that do not fulfil the inclusion criteria will be excluded.

#### Patients

To be eligible for participation in the study, patients must be 18 years of age or older, diagnosed with type 2 diabetes mellitus (International Classification of Diseases, Tenth Revision (ICD-10), codes E11 to E14) and enrolled in the DMP Diabetes program. Furthermore, they must be diagnosed with at least two ‘severe chronic comorbidities’ according to the definitions enshrined in German legislation [22]. These comorbidities include, but are not limited to, atherosclerosis (ICD-10 code I70), chronic coronary heart disease (ICD-10 code I25), chronic obstructive lung disease (ICD-10 code J44), asthma (ICD-10 code J45), cerebrovascular diseases (ICD-10 codes I60 to I69), depression (ICD-10 codes F32 and F33), heart failure (ICD-10 code I50), Parkinson disease (ICD-10 code G20) and/or chronic pain (ICD-10 code R52). Additionally, written informed consent is a prerequisite for participation in the study.

Patients who do not fulfil the inclusion criteria will be excluded. Additional exclusion criteria are severe acute psychiatric disorders (for example, schizophrenia, schizotypal and delusional disorders (ICD-10 codes F20 to F29)); dementia (ICD-10 codes F00 to F03); mental and behavioural disorders due to psychoactive substance use (ICD-10 codes F11 to F16, F18 and F19), except for alcohol (ICD-10 code F10) and tobacco use (ICD-10 code F17); malignant neoplasms (ICD-10 codes C00 to C97) and/or current chemotherapy or radiotherapy; transplanted organ and/or tissue status (ICD-10 code Z94); care involving dialysis (ICD-10 code Z49); insurmountable language and communication problems; and emergent cases.

#### Patients’ informal caregivers

One of the secondary research objectives is to assess the costs of informal care in both study groups (PTC vs. TAU). This information will be collected with the financial family burden (FFB) questionnaire (Table 1), which addresses one family member who provides the most care for the patient with type 2 diabetes. A *caregiving family member* is defined as the family member who (1) from the patient’s perspective, spends the most amount of time providing informal care (for example, a spouse, partner, father, mother, son, daughter or others) and (2) lives in the same household as the patient. It is important to note that it is up to the individual patients to identify, from among their social relationships, the family member who provides the most care. As such, this construct explicitly considers patients’ views and does not apply to an external definition. One informal caregiver per patient will be recruited.

**Table 1:** **Overview of outcome parameters, co-variables and instruments of the GEDIMAplus trial**^**a**^

**Outcome parameters**	**Measurement instruments**	**Data sources**	**References**
			
	Primary outcome		
Diabetes self-care	Summary of Diabetes Self-Care Activities Measure–German version (SDSCA-G)	PQ	[9] (our own translation, unpublished)
	Secondary outcomes		
Glycosylated haemoglobin A1c	Patient chart	PhysQ	–
(Severe) symptomatic hypoglycaemia	Patient chart/self-developed items	PhysQ	–
Health-related quality of life	EuroQol (EQ-5D)	PQ	[23]
Chronic disease–related self-efficacy	Self-efficacy scale (SES6G)	PQ	[24]
Health-care utilization and costs	Client Socio-Demographic and Service Receipt Inventory (CSSRI)	PQ	[25]
Costs of informal care provided by designated family member	Financial family burden (FFB) questionnaire	FQ	[26]
	Covariables		
Type of diabetes therapy	Patient chart	PhysQ	–
Comorbidities	Patient chart	PhysQ	–
Medication	Patient chart	PhysQ	
Perceived quality of care	PACIC-S	PQ	[27]
Emotional distress	PAID Short	PQ	[28]
Depression	WHO-5	PQ	Psychiatric Research Unit, WHO Collaborating Centre for Mental Health, Frederiksborg General Hospital [29]
Alcohol consumption	AUDIT-C (German version)	PQ	[30]
Migration status	Recommended ‘basic set of indicators for mapping migrant status’ in Germany	PQ	[31]
Sociodemographic data (sex, age, housing, partnerships, education, occupation, equalized household income)	Sociodemographic Standards of the Federal Department of Statistics (partially adapted)	PQ	Federal Office for Statistics [32]
Subjective social status	One-item standardized measure	PQ	German General Social Survey (ALLBUS) [33]

^a^AUDIT-C, Alcohol Use Disorders Identification Test; FQ, Family member questionnaire; PACIC-S, 11-item Patient Assessment of Chronic Illness Care short form; PAID Short, Problem Areas in Diabetes scale (short version); PhysQ, physician-reported measure captured by physician questionnaire/chart review; PQ: Patient questionnaire/patient-reported measure; SES6G, Self-Efficacy for Managing Chronic Disease 6-item Scale, German version; WHO-5, 5-item World Health Organization (Five) Well-Being Index.

### Recruitment

#### Study centres

This multicentre RCT will be conducted in 30 PCPs that formerly participated in a pilot study. PCPs were invited to participate in the study by sending an official letter from the study central office and requested their written consent for participation.

#### Patients

The 30 PCPs will receive from the study central office (through the intervention coordination centre, Genossenschaft Gesundheitsprojekt Mannheim (GGM)) a list of the inclusion and exclusion criteria for patients, along with a screening list with random numbers. PCPs will be asked to create a list of all potentially eligible patients, based on the inclusion and exclusion criteria, who are registered in their practice’s electronic database. In a next step, the PCPs will select 40 patients from among those on the list according to the sequence of random numbers. Randomly selected patients will be contacted and asked by the PCP physician whether they wish to participate in the study. Patients who are not willing to participate will be asked to give a reason for their decision (on a voluntary basis). The number of patients ‘screened’, ‘asked’ and ‘asked and not included’, as well as the reasons for exclusion or nonparticipation, will be documented by the PCP physician and reported to the study central office. This procedure will be repeated until a total of 20 patients per PCP are recruited. Patients who are willing to participate will be informed by the PCP physician about the aims, content, privacy issues and risks related to study participation. Patients who provide their informed consent to participate in the study will undergo a clinical examination and receive the patient questionnaires used to capture the PROs.

#### Patients’ informal caregivers

Patients who give their informed consent to participate in the study will be asked to hand over (on a voluntary basis) an information leaflet, an informed consent form and a questionnaire in a closed envelope to the family member who provides them with the most care. The participation of the ‘chosen’ family member is completely voluntary and independent from the participation of the patient who ‘chooses’ the family member.

### Randomization

Patients will be randomly allocated to care management (PTC) or usual care (TAU) at the individual level at a ratio of 1:1 stratified by (1) type of medical treatment (insulin vs. oral medication or no medication) of their index disease (type 2 diabetes) and (2) study centre (PCP). We previously identified the type of medical treatment as the strongest predictor of diabetes self-care in an unpublished scoping review and will thus capture this variable by a physician-reported questionnaire to serve as a validated stratification criterion. We will hence perform stratified block randomization with variable block lengths to ensure study groups of approximately equal size. In order to ensure concealment, the block sizes will not be disclosed. Instead, this specific information will be provided in a separate document with access restricted to the randomization authority only. The randomization procedure is closely linked to the data collection procedure insofar as that it will be performed centrally using the ICW Care Manager platform (InterComponentWare AG, Walldorf, Germany). All eligible patients participating in the study will be registered in this database, along with the physician-reported information about eligibility criteria and type of medical treatment of diabetes, during a 3-month recruitment period. ICW Care Manager contains virtual folders for the two groups (PTC and TAU) within each participating PCP. A randomization authority (that is, a designated person not involved in care of the trial patients and analysis of data) will access the database of registered patients in weekly intervals to monitor the recruitment process. Recruited and registered patients who fulfil the inclusion criteria will be centrally randomized (stratified by type of medical therapy and PCP) to the PTC or TAU arm using a computerized random number generator to create randomization lists for each stratum. The allocation will be documented separately in an Excel file (Microsoft, Redmond, WA, USA) with restricted access, except for the randomization authority. Patient allocation to either the intervention or control group will remain completely concealed until data collection related to primary and secondary outcomes at T0 is completed. After baseline data collection is completed, PCPs will be informed about the allocation of their patients (identified by unique study identification number) in an official letter from the intervention coordinating centre (GGM), which in turn will receive the information from the study coordinating centre (AMV).

#### Intervention

The intervention is a complex care management program [34] aimed at improving diabetes self-care behaviour among patients with type 2 diabetes and multiple comorbidities. The development of the multifaceted intervention (Figure 1) is based on (1) a 12-month pilot study (Gesundheitsbegleitung Diabetes Mannheim (GEDIMA)) conducted by the coordinating centre (GGM), including 171 patients; (2) focus groups with PCP physicians and specialist care providers involved in diabetes care; and (3) the active engagement of local patient self-help groups in formulating assessment contents and identifying community resources. The complex intervention will be delivered as an add-on to treatment as usual and will consist of three main elements: assessments, symptom and needs monitoring and digital structured blood glucose testing.

#### Assessments

At the beginning of the study, patients in the PTC group will undergo an IT-based assessment of medical and nonmedical needs and resources, which employs a structured protocol, administered by a PCPnetwork-based HCA who functions as the PCPnetwork case manager (NCM). The NCM is authorized to access the ICW Care Manager database with a unique password and username. At the beginning of the study, the NCM will have access to only anonymized data of those patients who are recruited into the intervention arm and are based at the PCP at which the NCM is in charge. The NCM will contact the PCPs in order to de-pseudonymize ‘her’ patients and to deliver the assessment. Note that the assessment results will not be used as efficacy outcomes of the intervention, but rather will be used solely for the purpose of delivering the intervention itself.

The contents of the assessment are demographics (age, sex, insurance status, living environment, education, migration status and/or cultural background), utilization of existing health and social services related to individual needs (participation in DMPs beyond DMP Diabetes, hospitalization in the previous 12 months, social services, home nursing services, medical aids, physiotherapy, podiatrist, psychotherapy, selected symptoms and/or clinical manifestations (thirst, lack of appetite, nausea, vomiting, dyspnoea, fatigue, wounds, disorientation), biomedical parameters (blood pressure, weight, height, blood sugar), SMBG, participation in diabetes education programs, problems in organization of care and social situation, tripping hazards and social resources (external caregivers, family, neighbourhood).

In addition, the NCM will perform the following standardized clinical assessments to identify entry points for individual support: Mini-Mental Status-Test, Lawton Instrumental Activities of Daily Living Scale (German version), tandem stand, depression (5-item World Health Organization (Five) Well-Being Index, German version) [29], mobility and/or physical activity, alcohol use (Alcohol Use Disorders Identification Test, German version) [30] and tobacco use (in pack-years). The ICW Care Manager platform provides the NCM access to physician-reported background information related to patient diagnoses, medications, HbA1c, proteinuria and potentially existing patient decrees (for example, living will, health-care proxy (‘Vorsorgevollmacht’), legal guardian (‘Betreuungsverfügung’)).The results of the initial assessment will be reported to a so-called dialogue assistant (DA) based at the PCP, who in turn will relay this information to the primary care physician in charge of the patient’s medical care. The assessment results will serve as the basis for setting common goals between the patient and his or her primary care physician, which will be based on the patient’s needs. The assessment results will be digitally stored on a secured server for later use by the NCM during follow-up home visits (FUPHV) and during the telephone monitoring. FUPHVs will occur 6 months (FUPHV1) and 12 months (FUPHV2) after study entry for patients in the PTC group (Figure 1).

Individualized assessments (including repeated reassessments) of medical and nonmedical needs by non-physician health-care workers have previously been reported to be an important element in attempts to improve diabetes-related outcomes such as HbA1c level, weight and body mass index, lipids, blood pressure, health-care service use and physical activity [34]. Further elements leading to improved outcomes are the use of decision-making algorithms by health-care providers and a focus on behaviour-related tasks [34], both of which are incorporated in the IT-supported assessment tool that will be deployed in this intervention. The focus on both medical and nonmedical needs ensures that the intervention is person-centred (not disease-centred) and thus congruous with evidence on how to improve diabetes care [14]. This type of assessment of patients’ needs is an outreach element which ensures that needs are assessed comprehensively and takes into account the individual patient’s social environment. The assessment is embedded in the process of primary care within the health-care system and mobilizes community resources to meet patients’ identified medical and nonmedical needs. By using this approach, we aim to reduce barriers to patients’ accessing support systems and medical and/or nonmedical services based in their local communities in order to improve management of their disease. There is evidence that the combination of these features is effective in improving diabetes care [14].

#### Symptom and needs monitoring

NCMs will engage in regular telephone monitoring using a structured list of questions. The content and frequency of the monitoring will be fixed, but the PCP and NCM may decide that additional items should be incorporated. The NCM will deliver at least a total of 24 telephone monitoring sessions according to a fixed schedule (every 2 weeks for a period of 6 months, then monthly for the rest of the intervention period for patients in the PCT group) (Figure 1). The items monitored will be new diagnoses; dyspnoea; incidence, frequency and severity of hypoglycaemia; blood pressure; weight; fatigue; medical and social problems; and follow-up on previously mentioned problems and hospitalizations since the last contact. The monitoring list was developed on the basis of experiences of GGM in prior studies (GEDIMA study, unpublished). The patient’s responses will constitute ‘red flags’ according to the urgency of the symptoms and signs. ‘Red flag’ answers require immediate contact with the PCP for discussion and to take steps to address them.

Evidence suggests that such high-intensity interventions (more than ten contacts) delivered for 6 months or longer are necessary in order to improve and sustain any change in self-care behaviour [35]. Application of this element in care management interventions in the German health-care context has been shown to be effective in the early diagnosis and treatment of acute complications and problems thereby reduce avoidable hospitalizations of chronically ill patients [6]. This monitoring will also be used as a feedback method related to patients’ control of their disease or behaviours, the results of which will be important in reinforcing positive behavioural change.

#### Digital structured blood glucose testing

Digital structured SMBG (using the ACCU-CHEK Smart Pix device reader and the ACCU-CHEK 360° View tool (Roche Diagnostics, Mannheim, Germany)) will be employed for symptom monitoring at 3-month intervals (a total of three times) (Figure 1). The NCM will collect the individual blood sugar profiles, transfer them into the ICW Care Manager database and report the profiles to the PCP in charge of the respective patient. The blood sugar profiles will serve as a basis for telephone coaching.

SMBG is an essential self-care element among patients with insulin-treated type 2 diabetes. However, for non-insulin-treated patients with diabetes, a synthesis of the existing evidence published by the German Institute for Quality and Efficiency in Health Care (IQWiG) revealed no evidence that SMBG improves diabetes-related outcomes [36]. As noted in the IQWiG report, though, this conclusion needs to be interpreted in light of scarce data, especially in terms of potential effects on the incidence of severe hypoglycaemia in non-insulin-treated patients [35]. The authors of a recent systematic review published by the Cochrane Collaboration, which included 12 RCTs (duration range from 6 to 12 months), wrote in their conclusion,

[W]hen diabetes duration is over one year, the overall effect of self-monitoring of blood glucose on glycaemic control in patients with type 2 diabetes who are not using insulin is small up to six months after initiation and subsides after 12 months. Furthermore, based on a best-evidence synthesis, there is no evidence that SMBG affects patient satisfaction, general well-being or general health-related quality of life. More research is needed to explore the psychological impact of SMBG and its impact on diabetes specific quality of life and well-being, as well as the impact of SMBG on hypoglycaemia and diabetic complications [36], abstract conclusions section.

The use of self-monitoring by patients with non-insulin-treated diabetes is controversial because reported estimates of benefit regarding reductions in HbA1c levels have varied in different systematic reviews [37]. A meta-analysis in which the researchers studied individual patient data also did not provide convincing evidence to support the routine use of self-monitoring of blood glucose by non-insulin-treated patients with type 2 diabetes [37]. The ‘scarce data argument’ and the potential effects of self-monitoring on the incidence of severe hypoglycaemia are the main rationale for including this element after T1 in the intervention for non-insulin-treated patients with type 2 diabetes. As such, contamination of primary endpoint effects due to participants’ use of the Smart Pix device reader will be avoided. Instead, by means of a secondary descriptive analysis, we hope that the study will add to the existing body of evidence regarding self-monitoring by non-insulin-treated patients with diabetes.

#### Composition and training of care management teams

The care management teams consist of 11 NCMs, 30 primary care physicians and 30 DAs based at each PCP. The task of the DA is to facilitate communication between NCMs, primary care physicians and patients. Each NCM will care for about 26 patients at about three PCPs. Some NCMs have previously participated in care management interventions related to diabetes and CHD (GEDIMA and Psychosocial Well-being and Disease Symptoms Health for Patients with Multi-morbid Coronary Heart Disease Patients (KHK-ProMA)) (*n* = 3); some have previously participated in care management interventions related to CHD alone (KHK-ProMA) (*n* = 2); and some have had no experience with care management programs (*n* = 6). One NCM is a native Turkish speaker who is specialized in the provision of diabetes education. This NCM will be responsible for delivering the care management intervention to patients with a Turkish background to overcome language barriers and appropriately address cultural preferences. We thereby aim to address the special needs of this social group, which constitutes about 22% of the population of Mannheim (as of 30 December 30), where the majority of study centres (PCPs) will be based. Potential language-related barriers to data collection will be countered by means of using a Turkish version of the patient questionnaire with this subgroup of participants.

Prior to the beginning of the intervention, all NCMs will be trained using a team-based curriculum. The curriculum was developed on the basis of experiences in the preceding pilot study (GEDIMA) and inputs from representatives of local patient self-help groups. Completion of a 32-hour team-based training course is mandatory for participating HCAs to function as NCMs.

#### Control

In the control group (TAU), practice teams will continue to provide standard care in the context of the PCP-centred care contract. This will involve gatekeeping for enrolled patients as well as regular training based on evidence-based guidelines through structured feedback in peer review groups. As population-based DMPs for diabetes and other selected chronic diseases are part of routine care in Germany, patients may voluntarily participate in these disease-specific DMPs. German DMPs consist of regular follow-up visits every 3 months or less. They include, on a regular basis and according to acute needs, clinical examinations, laboratory tests (for example, HbA1c tests), patient education and referrals to specialists (for example, ophthalmologists, cardiologists, nephrologists, neurologists) [12]. However, essential elements of care management interventions such as individualized assessment, care planning (including nonmedical aspects), home visits and frequent (symptom) monitoring are not routinely part of DMPs [6].

### Fidelity to the study protocol and variability between sites and care management teams

The intervention seeks to ensure fidelity to the study protocol with regard to adherence to the elements of the intervention and the timeline (Figure 1). The IT-supported delivery of the intervention allows assessment of fidelity to the main intervention elements for all participants in the intervention group. However, the intervention is explicitly designed to allow adaptations to local contexts and individual patients’ needs, preferences and cultural backgrounds with regard to the consequences of structured assessment and telephone monitoring. Adaptations to local contexts and individual preferences (for example, differential use of community resources and services) will be documented in the ICW Care Manager database by the NCM during her routine contacts. Figure 1 illustrates all elements and the timeline of the intervention.

### Data collection

AMV is responsible for administration, coordination, data management and monitoring (including database setup and validation, data entry, coding and query management). The intervention coordinating centre (GGM) will carry joint responsibility for administration, validation and data entry. Each patient will be asked by the recruiting physician to fill in a pseudonymized, paper-based questionnaire and hand it back to the recruiting physician in a closed envelope. This patient questionnaire will be used to capture the PROs analysed in this study.

Primary care physicians will document additional data from each patient’s charts by completing a paper-based questionnaire (including inclusion and exclusion criteria, diagnoses, medications, hospitalizations and hypoglycaemia prior to recruitment) and assessing the patient’s clinical status (for example, blood pressure, blood glucose, latest HbA1c value). The physician questionnaire will not contain any data that allows identification of the patient. The patient questionnaire and physician-reported chart review will be performed at T0, T1 and T2. In addition, in order to capture the cost of informal care for the health-care economic evaluation, patients will be asked to hand over the FFB questionnaire (in a closed envelope along with additional background information) to their relative who provides them with the most care for their diabetes. Data collected from the patient’s informal caregiver will be anonymized in a way that will allow linking the informal care burden with the disease severity of the respective patient. The collected data will be directed to the study central office (AMV) through the following channels:

1. Physician-reported chart reviews and patient questionnaires will be sent by the PCPs in paper-based format to the study coordination centre (GGM) by mail. At GGM, an authorized employee will enter the chart review data into the ICW Care Manager database and thereby register participating patients by study identification number. All data entered into the ICW Manager database will be transferred (encrypted via the internet) to the Centre for Information and Medical Technologies (Zentrum für Informations und Medizintechnologie (ZIM)) at the University Hospital Heidelberg and stored on a secured server. Authorized clinical monitors based at AMV can access the stored data in ‘real time’ to check conformity with inclusion criteria and monitor the progress of the recruitment process. Validation of entered data will be carried out by built-in checks in the ICW Care Manager and by clinical monitors.

2. The paper-based patient questionnaires containing the chart reviews and PROs will be stored at the GGM office and sent to the study central office (AMV) at regular intervals.

3. The FFB questionnaire designed for patients’ relatives will be sent directly to AMV by patients’ relatives in enclosed, post-paid envelopes. The written consent forms for patients’ relatives will be returned to GGM.

Patient questionnaires and FFB questionnaires will be scanned and digitally stored on a secured server at AMV.

### Monitoring

A risk-adapted strategy will be employed to ensure adherence to the portion of the study protocol dedicated to data collection and legal and ethical aspects. Authorized employees at the study central office (AMV) will conduct practice visits at 10% of PCPs randomly selected from among the pool of participating PCPs during the first 3 months of the intervention. These monitoring visits will include (1) an inspection of informed consents of all patients recruited by the PCP and (2) a review of the charts of 10% of patients recruited by the PCP to check if documented items and source data are congruent and that they are being recorded in conformity with the study protocol. A detailed monitoring manual will be developed by the study central office prior to the beginning of the intervention. Depending on the results of these visits, further quality assurance measures may be defined by the study central office (AMV) and the intervention coordinating centre (GGM).

### Outcome measures

#### Primary outcome

The intervention is aimed primarily at improving ‘self-care’ as a multidimensional construct consisting of the following five dimensions: diet, exercise, SMBG, foot care and smoking behaviour. This primary outcome is a PRO and will be operationalized by the German version of the revised Summary of Diabetes Self-Care Activities (SDSCA-G) [9]. The English version of the revised SDSCA is a widely used, appropriate [38], valid and reliable tool with moderate test–retest reliability [9] for capturing self-care behaviour.

The SDSCA-G is based on a structured forward–backward translation of the original English instrument. The SDSCA-G consists of 11 items and will be employed as part of the paper-based questionnaire. The response options for SDSCA-G items 1 to 10 (excluding smoking) are ‘days per week’ (on a scale of 0 to 7) on which the patient performed particular self-care tasks [38]. For each group of items (for example, diet), a mean number of days is calculated. The SDSCA-G scale is used as a continuous measure to determine if there has been improvement compared to baseline at subsequent time points. As a result of strong ceiling effects, the ‘medication’ item is not included in the most recent recommended version the instrument [9].

No score cutoff point on the SDSCA has been determined to discriminate between adherence and nonadherence [38], and no minimal clinically relevant change in SDSCA ‘scores’ has yet been formulated [39]. This means that, in order to determine a minimal clinically relevant change for this study, the research team will need to draw upon (1) published data on the degree of change in SDSCA values reported in interventions designed to improve self-management and (2) estimates of ‘realistic’ changes that the intervention can bring about.

The primary outcome that will be used to assess the efficacy of the intervention is the difference in mean change in SDSCA-G ‘score’ on items 1 to 10 (excluding smoking) per patient compared to T0) after 9 months of the intervention (T1) between both groups (PTC vs. TAU), determined on the basis of patients’ self-reports (Table 1).

#### Secondary outcomes

To address the secondary objectives of the study, we will attempt to capture the following secondary outcomes (Table 1):

1. The mean change in SDSCA-G ‘score’ on items 1 to 10 (excluding smoking) for each group (PTC vs. TAU) will be compared to T0 after 18 months of the intervention T2 to assess sustainability of effects with reduced intensity of care management and enhanced by SMBG.

2. The mean change in each of the five dimensions of self-care captured using the SDSCA-G instrument will be compared to T0 for each group (PTC vs. TAU) at T1 and T2 of the intervention to assess the influence of the intervention on specific self-care behaviours.

3. The mean change in physician-reported HbA1c (%) at T1 and T2 will be compared to T0 among the subgroup of patients with a T0 value of HbA1c less than 7.5%. A 10% improvement from the T0 value [39] at 9 months and 18 months will be regarded as a clinically relevant change.

4. The cumulative incidence of physician-reported (severe) symptomatic hypoglycaemia at T1 and T2 among those patients at risk for this event during the respective observation period will be evaluated. *Symptomatic hypoglycaemia* refers to hypoglycaemia with blood glucose less than 60 mg/dl, and ‘severe symptomatic hypoglycaemia’ will be considered an event of hypoglycaemia which necessitated external support. Patients will be considered to be ‘at risk’ for (severe) symptomatic hypoglycaemia for the subsequent time period if they receive oral antidiabetic medication and/or insulin therapy at T0 or T1.

5. The mean change in patient-reported HRQL will be captured by using the EuroQol instrument (EQ-5D) at T1 and T2 and compared to T0. EQ-5D is a 5-item, simple preference–based index measure which has frequently been applied to measure HRQL in type 2 diabetes patients (including the United Kingdom Prospective Diabetes Study). A difference of 0.03 on overall EQ-5D index score after 9 months and 18 months will be considered clinically relevant [39].

6. The mean change in patient-reported self-efficacy will be captured using the Self-Efficacy for Managing Chronic Disease 6-item Scale, German version, a short and validated instrument [24].

7. The equity/efficacy ratio will be calculated as the ratio between the change (compared to T0 at T1) in SDSCA-G ‘score’ on items 1 to 10 (excluding smoking), HbA1c (%) and EQ-5D index score among the lowest vs. highest socioeconomic patient subgroups (including education, occupation, income and subjective social status) in both arms of the trial (PTC and TAU). This includes subgroup analyses for migrant populations.

8. Further secondary outcomes will be captured using the Client Socio-demographic and Service Receipt Inventory (CSSRI) [25] and the FFB questionnaire [26]. Both instruments have been adapted to the special needs of patients with type 2 diabetes and were pretested before general application. CSSRI provides data on all kinds of service utilization and thus will be a crucial prerequisite to capturing resource consumption. In addition to direct and indirect costs, particular attention will be focused on the financial burden of informal care. Together with intervention costs, economic evaluations will be analysed from a societal perspective.

Table 1 provides an overview of all instruments that will be used in this study to capture primary and secondary outcomes as well as relevant covariables.

### Statistics

#### Sample size calculation

Sample size will be calculated based on the expected difference between the two treatment groups in mean change in the SDSCA-G score from T0 compared to T1. On the basis of data derived from published studies in which the researchers used the revised SDSCA score as an outcome measure [40-42], we estimate a mean change of 0.5 days (standard deviation = 2.0) in the overall SDSCA-G ‘score’ (calculated as the sum of days of items 1 to 10 divided by 10) per patient in 9 months as the minimal clinically relevant change. On the basis of these estimates, we will need a total of 506 patients (253 per arm) to detect an effect size (Cohen’s *d*) of 0.25 between groups (PTC vs. TAU) (50% relative increase in self-care) with a power of 80% by two-sided *t*-test of two independent samples at a significance level of 5%. Assuming a dropout rate of 15% over the period of 9 months, the overall sample size required is a total of 582 participants (291 per arm).

#### Analysis plan

A detailed description of the statistical methods that we will employ in this study will be provided in a statistical analysis plan. Data analysis will be done blinded to treatment arm allocation (that is, the treatments will be identified as 1 and 2 until analysis is completed). The primary analysis will be based on the 9-month follow-up data (T1).

#### Populations for analysis

The intention-to-treat (ITT) population will consist of all randomized patients. Following the ITT principle, patients will be analysed in the treatment arms to which they were originally randomized, regardless of whether they refused or discontinued treatment or whether other protocol deviations are known to have occurred. The per-protocol (PP) population will consist of those ITT patients with no major protocol violations. The criteria for the exclusion of patients from the PP population will be determined by the study team at the latest time point before database lock.

#### Statistical hypotheses, methods and analyses

The primary efficacy endpoint is the change in SDSCA-G score from T0 to T1, that is, the difference in SDSCA-G score from T1 to T0. The study objective will be statistically formulated as a test of the null hypothesis H_0_: μ_1_ = μ_2_ (mean difference in SDSCA-G scores between T1 and T0 in the two groups are equal) against the alternative hypothesis H_1_: μ_1_ ≠ μ_2_ (mean difference in SDSCA-G scores between T1 and T0 in the two groups are different). The null hypothesis will be tested at the two-sided significance level of α = 0.05.

In the primary efficacy analysis, we will use a multilevel regression approach with patients at level 1 and NCMs at level 2. The primary model will include treatment group as a fixed factor, NCMs as a random factor and the baseline value of SDSCA-G and type of medical treatment as covariates. The results will be presented as the mean between-group difference in SDSCA-G scores (T1 − T0) with the corresponding 95% confidence interval. The associated Cohen’s effect size *d* will be calculated. To support the primary analysis, all potentially relevant baseline characteristics will be added to the model as covariates in sensitivity analyses. A further sensitivity analysis of the primary endpoint will include an unadjusted two-sample *t*-test of the change in SDSCA-G score from T0 to T1. The results of these sensitivity analyses will serve to explain and interpret the results of the primary analysis. The primary analysis will be performed in accordance with the ITT principle. An additional sensitivity analysis will be conducted on a per-protocol analysis set.

In the statistical analyses of the secondary endpoints numbered 1 to 6 (see above), we will use the same multilevel approach that we will use in the primary analysis. According to the scaling of the considered endpoint, a linear or logistic two-level regression model will be fitted. The results will be presented in an analogous manner to the primary analysis. All statistical tests will be two-sided at the significance level of α = 0.05.

The equity/efficacy ratio [43] (and respective 95% confidence interval) will be calculated as the ratio of effects at T1 on health outcomes (change in SDSCA score, HbA1c (%) and HRQL) between the highest and lowest socioeconomic groups (related education, income, subjective social status) for both groups (PTC and TAU) compared to T0. Additional consideration of social group sizes will be ensured by calculating the concentration index (PTC vs. TAU) and plotting concentration curves at T0 and T1, respectively.

Subgroup analysis for nominal social groups will be performed by means of interaction terms [44] for migration status (Turkish migrants, non-Turkish migrants, participants without migration background) to be included in the regression equations for respective health outcomes at T1 (change in SDSCA score, HbA1c (%), HRQL).

Statistical analyses related to secondary outcomes 8 and 9 (see above) will be focused on the incremental cost-effectiveness ratio [45,46], which will be used to relate mean cost differences to mean differences in benefits between the intervention and control groups. The bootstrap method will be used to quantify statistical uncertainty, and a net benefit approach will be applied [47,48]. Maximum willingness to pay thresholds and cost-effectiveness–acceptability curves [49,50] will be determined. Sensitivity analyses will provide information on robustness of assumptions.

Because no adjustments for multiple endpoints are planned, the findings, especially those related to secondary outcomes and subgroup analyses [44], will be interpreted with caution in view of the number of statistical tests undertaken. Only the results of the primary efficacy analysis will be interpreted in a confirmatory manner. Confirmatory subgroup analyses are not planned. No interim analysis with regard to efficacy will be done.

#### Missing data

In cases of missing data, multiple imputations will be used to complete the data set for analysis. A complete case analysis will be performed to assess the influence of missing data and the value of adopting multiple imputation methods.

### Process evaluation

Data collected during NCM-led assessments and delivery of the intervention will be exported from the ICW Care Manager in anonymized format to perform a process evaluation. This evaluation will include data related to participation in DMPs, hospitalizations, use of social services, existence of patient advance directives, clinical parameters (blood pressure, body weight, height, blood sugar), use of podiatrists, use of psychotherapy, participation in diabetes education, results of clinical assessments and actions taken.

### Ethics and legal aspects

The study is being conducted in accordance with medical professional codex and the Helsinki Declaration (2013). The study is also being carried out in accordance with the German Federal Data Security Law (BDSG). All professionals participating in the study oblige to adhere to the above-mentioned declarations and laws. Participation of PCPs, patients and/or their relatives is voluntary. Consent can be withdrawn at any time without any consequences regarding patients’ (usual) care. All patients will be informed about the aims, content, duration and process of the trial, particularly with regard to risks and unintended consequences, through written information brochures and face-to-face communication with their PCP physicians. Data will be collected and analysed pseudonymously for patients, patients’ relatives, physicians and NCMs. Data obtained from DAs will be anonymized. Pseudonyms will be generated as follows. PCPs will be numbered 1 to 30, and the key will remain in the intervention coordinating centre (GGM). Patients recruited within each PCP will be given a pseudonym consisting of a combination of the PCP number and a running number (for example 3-15 for patient 15 in PCP 3). The key will remain in the recruiting PCP. Patients’ relatives will be given pseudonyms consisting of the letter ‘R’ plus the associated patient’s pseudonym (for example, R-3-15 for the relative of patient 15 in PCP 3). No key will be generated for patients’ relatives. NCMs will be numbered from 1 to 11. The key will remain in the intervention coordinating centre (GGM). Access to all keys will be forbidden for anyone except the key holders.

Special emphasis in the information brochure will be paid to the following privacy issue. During data entry of physician chart reviews into the ICW Care Manager database at T1 and T2, the authorized employee at GGM will have access to electronic patient records data, including full names, dates of birth and assessment results from the preceding time period since T0 for all patients in the intervention arm. This means that patients’ identity will not be concealed during data entry at T1 and T2. For the data analysis, however, all data will be fully anonymized when exported by the authorized data manager at the study central office (AMV). This privacy issue will be handled as follows: (1) through the written informed consent form, in which we explicitly mention that the patient’s identity may be disclosed during data entry at T1 and T2, and (2) through a written declaration of confidentiality, which must be signed by the authorized employee of GGM in order to qualify for data entry.

Patients’ relatives will be informed about the aims, content, duration and process of the trial, particularly with regard to risks and unintended consequences, through written information brochures. Written informed consent will be obtained from patients’ relatives. Further privacy issues are related to the hardware and software infrastructure that will be employed in this study within the scope of the ICW Care Manager database. To address these issues, the software will be used on a secured server at ZIM.

The system architecture for this project draws upon four virtual servers: an application server and database server for the production environment and an application server and database server for the test environment. A general proxy server will precede these servers to ensure the security of data stored on the database server. Access to the application-server via the internet will be necessary for the NCM to conduct the intervention, and physician-reported data entered at T0 to T2 by the GGM employee will also be transferred via the internet. Both external communication (proxy server with the internet) and internal communication (proxy server with the application server) will be secured via HTTPS. The ICW Care Manager system uses TLS 1.1 encryption to guard against unauthorized access to protected health information that is transmitted over an electronic communications network.

Control of access to the application will be guaranteed by person/entity authorization with a username and password combination. Passwords will be salted with a 128-bit salt and hashed using a cryptographically strong mechanism. Password complexity will be enforced by the application according to the password complexity configured by the customer. Control of access in the application will be ensured by user administration, which allows for the definition of roles and permission to restrict functionality (read, write, create, update and delete) on a graphical user interface (GUI) level and to restrict access to data objects. Patient-level security is guaranteed by restricting the visibility of patient identifying information and all patient-related data to a user or a group of users (for example, to guarantee that NCMs have access only to digital records of ‘their’ patients in the intervention am, but not to the patient records of other NCMs or to the control group records). Audit control will include a GUI-based audit feature which allows a user with the relevant permissions (for example, the authorized clinical monitor) to query an audit log that contains entries of all relevant accesses to the system with specification of the accessing user, date, time, type, scope and accessed data object. Furthermore, through technical file-based logging, the application itself logs technical information in disk-based log files that may contain relevant information such as system startup, shutdown time stamps and import processes.

The study protocol was approved by the ethics committee of the Medical Faculty of the University of Heidelberg (S-590/2013) and by the ethics committee of the Medical Association Baden-Württemberg (B-F-2014-007) prior to the start of the study. The trial is registered with Current Controlled Trials (ISRCTN 83908315).

## Discussion

We hope that this study will contribute to current knowledge about the efficacy and feasibility of PCPnetwork-based approaches to handling the complex health-care needs of patients with type 2 diabetes and multiple comorbidities. Delivering person-centred, comprehensive care while taking into account and mobilizing community resources [14] is a challenging task for health-care systems. Care management interventions at the primary care level in the German health-care system have previously been evaluated, with promising results [6,17-19]. However, care management has turned out to be particularly challenging to deliver for small PCPs. PCPnetwork-based approaches might be a solution in this context, because they allow the provision of intensified care to patients by several practices, thus reducing the workload of smaller PCPs. Provided that the intervention proves to be efficacious, this care program could be disseminated throughout PCP-centred care contracts.

## Trial status

Recruitment of the study centres has been completed. Recruitment of patients started on 1 February 2014 and was completed in May 2014.

## Abbreviations

AMV: Department of General Practice and Health Services Research, University Hospital Heidelberg; DA: Dialogue assistant; FUPHV: Follow-up home visit; GGM: Genossenschaft Gesundheitsprojekt Mannheim; HCA: Health-care assistant; HRQL: Health-related quality of life; ICW: InterComponentWare; IQWiG: Institute for Quality and Efficiency in Health Care; IT: Information technology; NCM: Network case manager; PCP: Primary care practice; PCPnetwork: Primary care practice network; PRO: Patient-reported outcome; PTC: Pathways to Change; SDSCA-G: Summary of Diabetes Self-Care Activities, German version; SMBG: Self-monitoring of blood glucose; TAU: Treatment as usual; TLS: Transport layer security.

## Competing interests

The authors declare that they have no competing interests.

## Authors’ contributions

KB, TF, WB, MM, JS, DO, COJ and HJS designed the intervention. RB and SS provided advice on the study design regarding the integration of technical components. JK, COJ, TF and MK made important contributions to the study protocol. KB drafted the first version of the study protocol and the manuscript. JK, COJ, MK, DO, SS and TF critically revised the manuscript for important intellectual content. All authors read and approved the final manuscript.
